# Differential role of CXCR3 in inflammation and colorectal cancer

**DOI:** 10.18632/oncotarget.24730

**Published:** 2018-04-03

**Authors:** Jessicca D. Abron, Narendra P. Singh, Angela E. Murphy, Manoj K. Mishra, Robert L. Price, Mitzi Nagarkatti, Prakash S. Nagarkatti, Udai P. Singh

**Affiliations:** ^1^ Pathology, Microbiology and Immunology, School of Medicine, University of South Carolina, Columbia, SC 29208, USA; ^2^ Department of Biological Sciences, Alabama State University, Montgomery, AL 36104, USA; ^3^ Cell Biology and Anatomy, School of Medicine, University of South Carolina, Columbia, SC 29208, USA

**Keywords:** inflammation, CXCR3, chemokine, colorectal cancer, tumor microenvironment

## Abstract

Chemokines (CXCR3) and their ligands (CXCL9, CXCL10, and CXCL11) exert exquisite control over T-cell trafficking and are critical for activation, differentiation and effector T cell function. CXCR3 is important for CD4 Th1 cells, CD8 effectors, memory cells, and for the function of natural killer and natural killer T cells. The presence of high cytotoxic CXCR3 ligand expression on CD8 T cells in colorectal cancerous tissue has been well documented in the past. CXCR3 and its ligands are differentially expressed at sites of inflammation and within the tumors. Further, the expression of CXCR3 and its ligands has been correlated with both the presence of effector T cells within tumor tissue and disease-free survival of patients. However, effector T cell infiltration into primary and metastatic tumors is highly variable and, in fact, often absent. Thus, understanding why T cells fail to infiltrate into tumors and determining the way to improve effector T cell entry into tumors would be important advances in efforts to harness the power of the immune system to fight cancer. To this end, the recent exciting discovery that CXCR3 is functionally expressed on regulatory T cells and also induces the differentiation of peripheral CD4 T cells into regulatory T cells, might address the novel clinically relevant question of the therapeutic potential of the CXCR3 system. This is also coupled with the fact that increases in CXCR3 expression also improves effector T cell function. This review describes the differential role of CXCR3 induction on peripheral and tumor microenvironment inflammation. Further, this review, tied with important findings from our laboratory, demonstrates that polyphenols induce CXCR3 expression on regulatory T cells and increases CXCR3 ligands in the tumor microenvironment, which act together to suppress colorectal cancer through a differential mechanism discussed herewith.

## INTRODUCTION

Chemokines are heparin binding chemotactic cytokines that regulate trafficking and position of cells by activating the seven-transmembrane G-protein-coupled chemokine receptors (GPCR). The target cell specificity of each chemokine is determined by the expression pattern of its cognate receptors. Chemokines are structurally divided into four subgroups, namely C, CC, CXC and CX3C based on the first two N-terminal cysteine residue [1]. Chemokines can be functionally classified as inflammatory or homeostatic based on their expression patterns after insult [2]. Inflammatory stimuli induce the expression of chemokines to induce cellular infiltration including T cells, granulocytes, and monocytes/macrophages. Till date, over 50 chemokines and 20 signaling chemokine receptors have been identified [3]. Chemokines not only coordinate the migration of immune cells, but also play an important role in maturation of immune cells and generation of adaptive immune responses [4]. Dysregulation of chemokines impacts a broad range of human diseases including autoimmune and inflammatory diseases [5]. Chemokines play an important role in complex tumor microenvironments (TME) made up by many different cell types. In the TME host and cancer cells release an array of chemokines, resulting in recruitment and activation of various cells to induce a pro-or anti-tumor response. To this end, the CXCR3 chemokine system received a lot of attention especially for differential function in both the TME and peripheral immune response. CXCR3 has been strongly associated with tumor progression in advanced colorectal cancer [6]. Further, CXCR3 has been considered as a potential therapeutic target for many inflammatory diseases and a few cancer models. In this review, we discussed the differential role of CXCR3 during peripheral inflammation and TME in colorectal cancer.

### CXCR3 family of chemokines

Chemokines can be defined as small (8–15 kD) proteins that induce chemotaxis, tissue extravasation, and modulators of the functional properties of leukocytes during inflammation. CXCR3 and its ligands CXCL9, CXCL10, and CXCL11 are from the non-ELR CXC chemokine group of structurally and functionally related molecules. CXCR3 is not detectable in normal conditions in most non-lymphoid tissue, but are strongly induced by the cytokine IFN-g during infection, injury and immuno-inflammatory responses. Thus, the CXCR3 family of chemokines is highly inducible by IFN-g and shares the ability to promote the directional migration of activated and memory, but not naïve T cells. Recently attention has been focused on the differential function of CXCR3 and its ligands on systemic inflammation in the TME. Even though CXCR3 is highly expressed on activated effector T cells in peripheral inflamed tissue, inhibition of CXCR3 pathways therapeutically for human disease has been unsuccessful so far. Further, in some murine models, global CXCR3 inhibition paints a confusing picture on the outcome of disease. To this end, previous studies have shown unique, but sometimes contradictory results, thus providing a strong rationale for more in-depth investigation in this area. Therefor in this review, we discuss the divergent roles of CXCR3 and its ligands on the induction of peripheral inflammation vs suppression of tumor number and size via different mechanisms in colorectal cancer associated with intestinal inflammation.

### Role of CXCR3-ligands in mediating inflammation and autoimmune diseases

CXCR3 ligands are CXC chemokines secreted by a variety of cells including endothelial, epithelial, T, natural killer (NK), natural killer T cells (NKT), fibroblasts, neutrophils, monocytes, and dendritic cells (DCs). As a group CXCR3 and its ligands have been extensively studied during inflammation and in autoimmune diseases [7]. CXCR3 ligands are chemotactic for leukocytes, as well as for activated T lymphocytes of the Th1 phenotype that express CXCR3 [8–15]. In addition, CXCR3 ligands can also compete with eotaxin for the binding of CCR3^+^ cells to inhibit migration and Ca^2+^ changes, thereby enhancing the polarization of Th1 cells and inducing inflammation [16]. CXCR3 ligands differentially induce chemotaxis efficiency during inflammation [17], through the initiation of IFN-g, which is associated with Th1 type immune responses [18]. In between three CXCR3 ligands, the C-terminal region of CXCL10 has a high affinity for surface heparan sulfate glycosaminoglycan (GAG), which is expressed on epithelial, endothelial, and hematopoietic cells [19, 20], as compared to other ligands. Our laboratory and others have shown that all three CXCR3 ligand expression is elevated in many autoimmune diseases [21], as depicted in part in Table 1. Further, in particular, CXCL10 expression has been shown to increase in periodontal and autoimmune liver diseases [22, 23]. CXCL9 is upregulated during Alzheimer’s [24] and Hodgkin’s diseases [25, 26], infection of the central nervous system [27], Grave’s disease [28], asthma [29, 30], and glomerulonephritis [31]. All of the CXCR3 ligands play a role in multiple sclerosis [27, 32], bronchiolitis [33], and skin/mucosal inflammation [34, 35]. We have shown that blocking CXCR3 ligands ameliorate colitis and interstitial cystitis severity through reduction of inflammatory Th1 cells from mucosal sites. Taken together, these studies suggest the importance of the CXCR3 chemokine family in initiation and progression of inflammation during infection and autoimmune diseases.

**Table 1:** Summary of CXCR3 ligands expression in various disease

Disease	Ligands	Receptors	Cell type effected	References
Autoimmune Liver disease	CXCL9/10	CXCR3	Hepatocytes	Nishioji K. *et al.* Clin. & Exp. Immunol. 2001; 123:271–9.
Alzheimer’s Disease	CXCL10	CXCR3	Astrocytes	Xia M.Q. *et al.* J. Neuroimmunol, 2000; 108:227–35.
Hodgkin’s	CXCL9/10	CXCR3	Hodgkin RS cells	Ohshima K. *et al.* Int. J. Cancer, 2002; 98:567–72.
Central Nervous System	CXCL9/10	CXCR3	T cells	Liu MT. *et al.* J. Immunol. 2001; 166:1790–5.
Grave’s	CXCL9/10	CXCR3	Glomerular	Romagnani P. *et al.* J. Pathol, 2002; 161:195–206.
Asthma	CXCL10	CXCR3	T cells	Medoff B.D. *et al.* J. Immunol. 2002; 168:5278–86.
Glomerulonephritis	CXCL9/10	CXCR3	Mesangial	Romagnani P. *et al.* J. Am. Soc. Neprol. 10; 2518–26.
Multiple Sclerosis	CXCL9/10/11	CXCR3	Astrocytes	Salmaggi A. *et al.* J. Inter. Cyto. Res. 2002; 22:631–40.
Bronchiolotis	CXCL10	CXCR3	Mononuclear	Belperio J.A, *et al.* J. Immunol. 2002; 169:1037–49.
Mucosal Inflammation	CXCL9/10/11	CXCR3	Keratinocytes	Flier J, *et al.* J. Pathol. 2001; 194:398–405.
Lupus	CXCL10	CXCR3	T cells	Rotondi M. *et al.* Endocr. Rev. 2007; 28:492–520.
Diabetes	CXCL10	CXCR3	T cells	Shiozawa F. *et al. Arthritis Res Ther*. 2004; 6:R78–R86.
Interstitial cystitis	CXCL9, CXCL10	CXCR3	T cells	Singh UP*. et al.* I. Immunol. 2003; 1:171(3):1401–1406.
Colitis	CXCL10	CXCR3	T cells	Singh UP*. et al.* Plos One 21; 8(11) e79751.

Summary of the various cell types in autoimmune diseases mediated by CXCR3 and its ligands.

### Role of CXCR3 and its ligands in colitis associated with colorectal cancer

It has been well established that colitis is induced, in large-part, by the infiltration of T cells, macrophages, and neutrophils that produce Th1 cytokines in the mucosa [36, 37]. Further, it has been shown that CXCR3^+^ T cells increase in the lamina propria (LP) of inflammatory bowel disease (IBD) patients as compared with normal healthy donors [38]. For the past 15 years our laboratory has worked on this chemokine and have shown that CXCR3 and its ligands are upregulated at sites of experimental colitis [39]. We have also shown that systemic CXCR3 ligands increase significantly in Crohn’s disease (CD) patients, as compared to normal healthy individuals [21]. In a similar study, we noticed changes in the intestinal pathology from patients with the highest levels of CXCR3 ligand expression, including heavy cellular infiltration that mainly constitutes by T cells, neutrophils and macrophages. Further, the colorectal histology of normal healthy individuals showed hypertrophied epithelial layers at multiple sites, with only a few inflammatory infiltrates mainly composed of lymphocytes, plasma cells and macrophages [21]. In particular, CXCL10 has received considerable attention in recent years and has been shown to be upregulated during colitis [40], while CD tissues have been shown to express another ligand, CXCL9, as well as CXCR3 [41–44]. We have shown that CXCL10 blockade ameliorates spontaneous experimental colitis [39], which is mediated predominantly by Th1-type αb TCR^+^ CXCR3^+^ cells [45]. We have also shown that anti-CXCL10 antibody treatment dramatically reduced IFN-g levels and other proinflammatory cytokines in mice during chronic colitis as compared to vehicle treated mice. However, CXCR3 and its ligands have been shown to increase during colitis, but the role that these chemokines play in pathogenesis, disease susceptibility and progression of colorectal cancer is not very clear.

Colitis is strongly linked to the development of colorectal cancer [46]. The etiology of the third most common form of cancer, colorectal cancer, is complex and involves many factors including genetic, immunologic, environmental influences, and presents a significant disease burden Worldwide. Biological determinants that influence the probability of developing [47] colorectal cancer can include, but are not limited to, immunological disorders associated with cellular or humoral responses. Interestingly, colitis patients have an increased lifetime risk for developing inflammation associated with induction of colorectal cancer [48, 49]. The literature unequivocally shows the importance of CXCR3 expression on Tregs, Teffs, and macrophages in colorectal cancer induction and progression. To this end, it has been shown that CXCR3 promotes colorectal cancer metastasis to lymph nodes [50].

**Figure 1:**
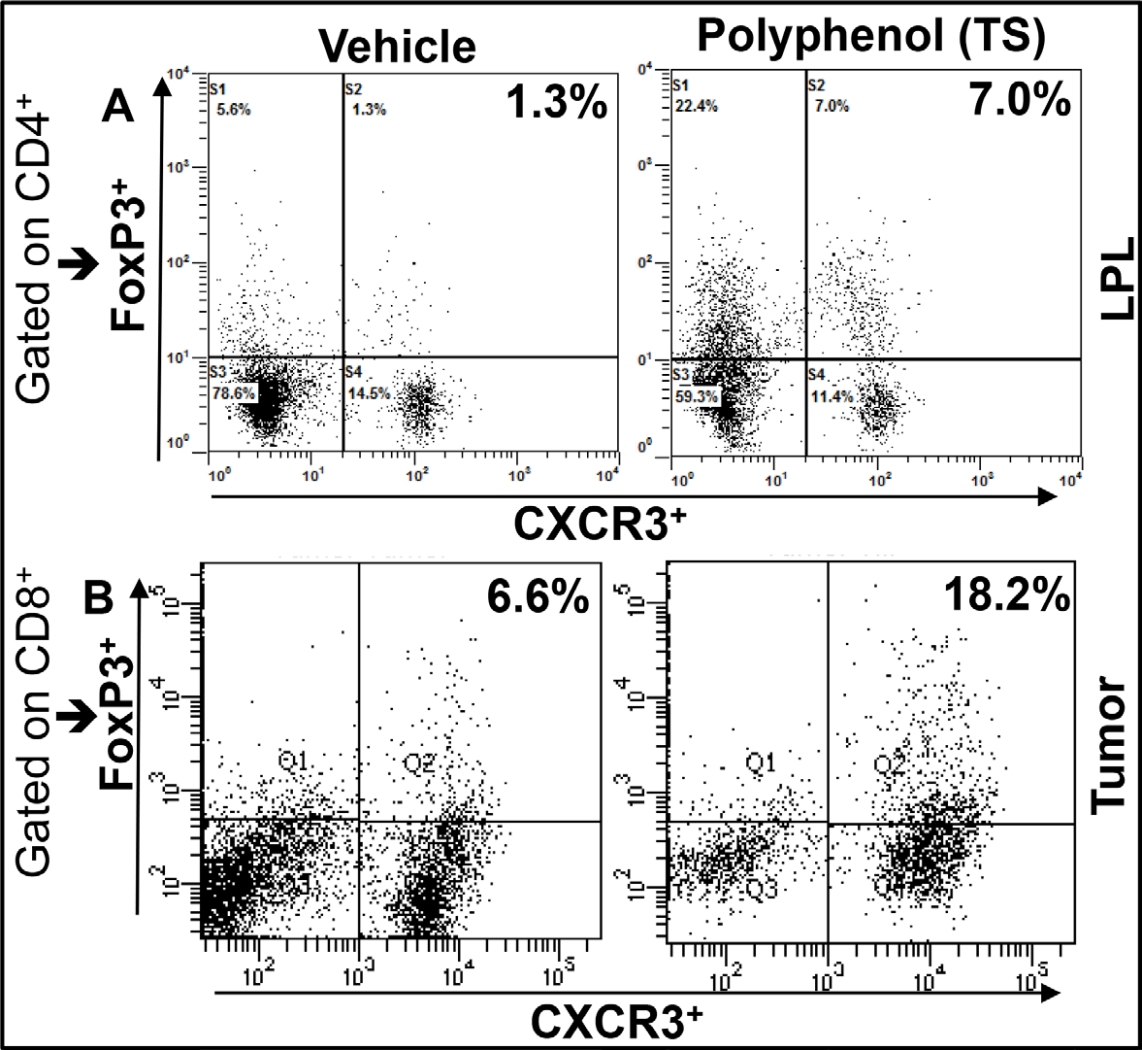
TS differentially induces the expression CXCR3^+^ Tregs in periphery and CRC Tumors IL-10 knockout mice on a C57BL/6 background were given 100 ml of vehicle or TS (100 mg/kg body weight) every 3 days by oral gavage from 8 weeks after the onset of symptomatic colitis through week 16 The cells from LPLs were stained for FoxP3 and CXCR3 gated on CD4 T cells, then analyzed by flow cytometry. Changes in the mean percentage of FoxP3+CXCR3+ cells are shown in upper right quadrants (**A**). Azoxymethane (AOM) and Dextran sodium sulphate (DSS) induced BL/6 mice were given similar dose of TS for three weeks and tumor were isolated and stained for Foxp3 and CXCR3 gated on CD8 T cells (**B**). Experimental groups consisted of 5 mice; experiments were repeated three times.

**Figure 2:**
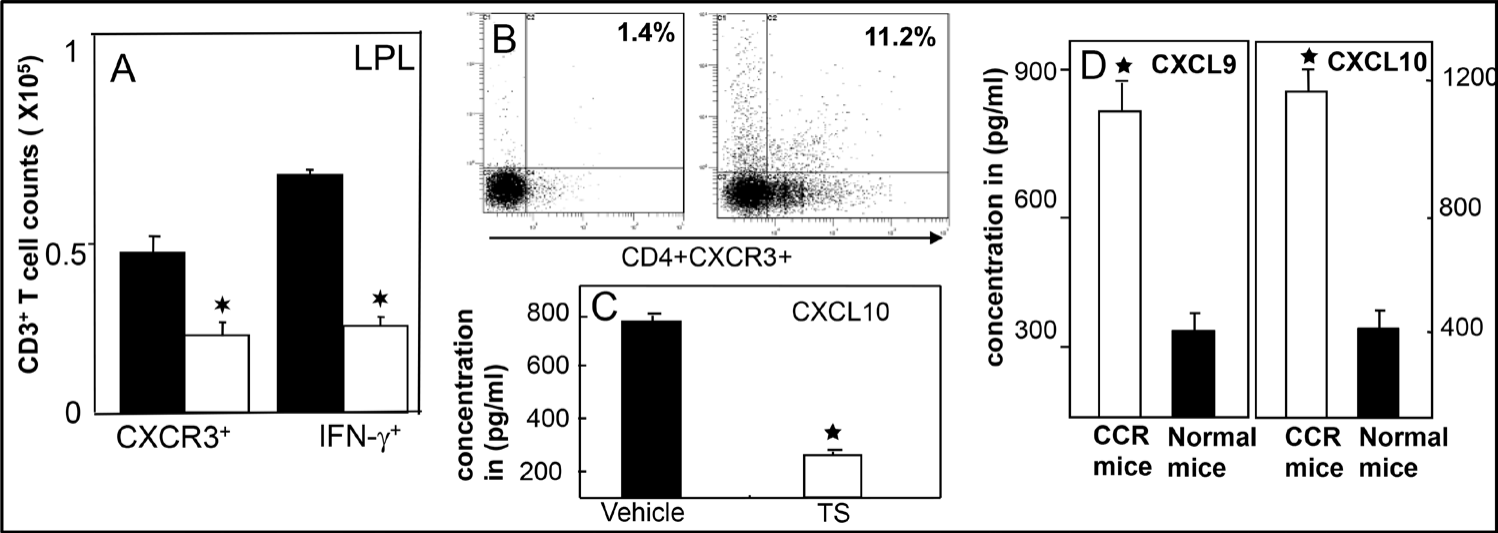
TS reduced systemic CXCR3 lignads and enriched in tumor infiltrating T cells and tumor microenvironment Lamina propiria (LP) lymphocytes and tumor cells were isolated from the Azoxymethane (AMO) and DSS induced BL/6 mice treated with vehicle (■), TS (⁪□) and changes in the numbers of CD3 T cells expressing IFN-g and CXCR3 were determined by flow cytometry and expressed as the total number of cells/mice ± SEM. Data shown are from a representative experiment; three independent experiments involving 6 mice/group yielded similar results (**A**). Panel (**B**) shows CXCR3 expression from Tumor of DSS induced CCR mice; (**C**) shows suppression of systemic CXCL10, a ligands for CXCR3 after TS treatment and (**D**) shows increase in CXCR3 ligands levels in tumor microenvironment in CCR mice. Asterisks indicate statistically significant differences (*p* < 0.01) between different experimental group.

We are excited by our recent finding that shows, polyphenol 3, 5, 4’ trihydroxy-trans-stilbene (TS) treatment induces CXCR3 expression on Tregs at peripheral sites in lamina propria lymphocytes (LPLs) and in TME as compared with vehicle-treated mice (Figure 1). This exciting data provides a wide-open opportunity to determine whether induced CXCR3 expression on Tregs by the natural TS compound: 1) Limits the effectiveness of peripheral (colorectal) CXCR3 inhibition; 2) Whether CXCR3 ligands released from antigen presenting cells (APCs) attract CXCR3^+^ Teffs to the TME; 3) Further activating CXCR3 ligands (CXCL9 and CXCL10) to eradicate tumor cells and suppress the severity of colorectal cancer. Further, in a similar study, we noticed that CXCR3 and its ligands are important during Teff entry into colon tumors in the Azoxymethane (AOM) and dextran sodium sulphate (DSS)- induced models of colorectal cancer (Figure 2). Interestingly, we noticed that TS reduced the level and expression of peripheral CXCR3, IFN-γ and CXCL10 (Figure 2A and 2C). In contrast TS also induces CXCR3 and its ligand level and expression in the TME of DSS-AOM induced colorectal cancer as compared to normal mice (Figure 2B and 2D). This is exciting data on TS induction of differential CXCR3 expression and function on peripheral versus tumor sites that might affect cancer progression. These exciting results have led us to believe that, TS-induced CXCR3 expression on CD8^+^Tregs contributes to dampening of the suppressive capacity of Tregs and facilitates more Teffs recruitment to tumor sites thus inducing Teffs function that reduces the tumor number and size. In contrast, TS induced CXCR3 expression of CD4^+^Tregs at peripheral sites suppress the immune response and systemic inflammatory cytokines, thus reducing inflammation and leading to effective suppression of both colitis and associated colorectal cancer.

### How CXCR3 ligands differentially mediate peripheral and tumor microenvironments

The control of T-cell migration is absolutely critical for the differentiation, activation and Teff function at inflammatory sites. In most cases, Teffs fail to infiltrate into certain tumors and for this reason, finding a way to increase Teffs entry into the TME would be an important advance to move the cancer field forward. It is well known that CXCR3 facilitates Th1 and Teffs recruitment to the site of inflammation and facilitates Tregs recruitment to the same site, thus limiting the effectiveness of CXCR3^+^Teffs function in anti-tumor immunity. This observation is coupled with impressive results by using adoptive cell transfer models where CXCR3-deficient Th1 and Teffs have impaired ability to enter into tissue and cause disease. The presence of tumor-infiltrating leukocytes is now considered to be an indication of the host immune response to tumor antigens [51, 52]. Various studies have suggested that host CD4 and CD8 cells can also control tumor growth [53, 54]. It is well known that Teffs can eliminate tumor cells, whereas Tregs suppress Teffs to dampen this response. Thus far, only a few immunotherapies, such as PD-1, have been used to attenuate Tregs suppressor function and reverse Teffs cell dysfunction to eliminate certain cancers [55, 56]. In a mouse cancer model, immunotherapy promoted the anti-tumor response by enhancing infiltration, proliferation, and CD4+CD8 Teffs activity [57]. The differentiation of naïve CD4 and CD8 into Th1 and Teffs can be maintained by sustained upregulation of CXCR3. We have shown that TS also activates Sirt1, reduces colitis-associated colorectal cancer, and inhibits tumor formation and size [58, 59]. Further, we have also shown that CXCR3 ligands are upregulated during colitis [45] and anti-CXCL10 treatment inhibits colitis symptoms [39]. Thus, it is safe to believe that CXCR3-expressing Tregs interact with antigen-activated CXCR3 ligands secreting APCs at the site of inflammation and identifying these APCs for Teffs suppression. However, at tumor sites, inhibition of CXCR3 expression on Tregs limits the effectiveness of Teffs recruitment and suppression of size and number of colorectal tumors. The current shift in paradigm on T cell based immunotherapy is crucial to improve the clinical outcome in colorectal cancer patients. Thus, innate and Teffs response can be successfully expanded by introducing a potent adjuvant in the tumor microenvironment [60]. CXCR3 expression on Tregs and Teffs in tumors represents an under investigated area and functional characterization of CXCR3 induction on Tregs and Teffs function in colorectal cancer needs further in-depth study.

### CXCR3 axis as a therapeutic option for both inflammation and colorectal cancer

The complex etiology of inflammation and colorectal cancer involves immunologic, genetic, and environmental factors as well as the intestinal microenvironment. To date, treatments available for inflammation and associated colorectal cancer can only reduce periods of active disease, but often bring marginal results and the disease becomes refractory. Therefore a safe, effective, non-toxic and low-cost prevention of inflammation and associated colorectal cancer is urgently needed. The literature on systemic CXCR3 inhibition in murine models of inflammation is contradictory and a therapeutic trial of CXCR3 blockade of inflammatory disease has been disappointing till date [61]. In an inflammatory condition CXCR3 is highly expressed at peripheral inflammatory sites and global inhibition of CXCR3 therapeutically in human autoimmune disease is not giving predicted results. To this end, results on infiltration of Teff in primary and metastatic tumors are highly variable. Recent studies in humans have emphasized the potential importance of CXCR3 expression on T cell infiltration into tumors and patient survival [62, 63]. The overexpression of CXCR3 might be implicated as a favorable prognostic biomarker for human advanced gastric cancer. CXCR3 ligand expression has been correlated with T cell infiltration status in colitis, metastatic melanoma, colorectal carcinoma and disease free survival [64]. Current immunotherapy targeting T cell checkpoints, such as programmed cell death (PD-1), are currently one of the promising and exciting new therapeutic approaches for cancer therapy [65]. PD-1 is an inhibitory immune receptor that is expressed on T cells after activation. The possible mechanism by which PD-1 works is by increasing the recruitment of non-exhausted T cells and enhancing Teffs function in tumor microenvironment [66]. This is clearly linked with increases in CXCR3 ligand expression and recruiting new Teffs to the tumor site as observed by others and in our study presented in this review. The notion of enhanced CXCR3 expression has been supported by a recent murine study that shows anti-PD-1 therapy increases CXCL10 expression in renal cell carcinoma tumors [65]. This suggests that CXCR3 might play a role in the efficacy of anti-PD-1 therapy. Further, a recent discovery shows that CXCR3 expression on Tregs plays a critical role in limiting the endogenous anti-tumor response as well as limiting the clinical efficacy of therapeutic anti-tumor response [67]. Thus recruitment of Tregs into tumors and their interactions with APCs within tumors, as well as recruitment and interactions of Teffs in the TME are crucial for cancer therapeutics. Further, CXCR3 ligands produced by APCs increase the frequency and duration of T-cells and dendritic-cells contacts in the TME. These findings and our preliminary results provide strong support that induction of CXCR3^+^ expression enables CXCR3^+^Tregs to license APCs to attract and activate CXCR3^+^ Teff function through cytokine (IFN-g) induction in the TME. This might be beneficial for colorectal cancer amelioration. In contrast, during inflammatory and autoimmune disease inhibition of CXCR3 and its ligands reduces the activated T cells and enhances Tregs in peripheral inflammatory sites to mitigate inflammation by reducing inflammatory cytokines. Thus, CXCR3 can be beneficial for both reducing systemic inflammation as well as for anti-tumor responses.

**Figure:**
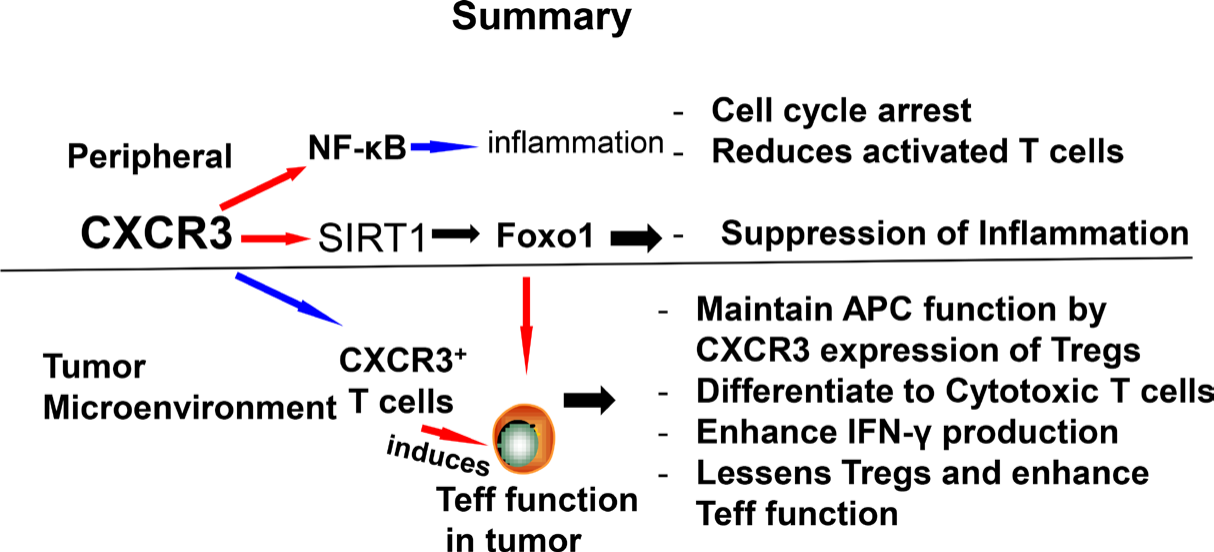
Schematic showing possible mechanism and differential role of CXCR3 in inflammation and colorectal cancer. The differential effects of CXCR3 by reducing peripheral inflammation and lessens Tregs function that enhances Teff function to mediate colorectal cancer.

## CONCLUSIONS AND FUTURE DIRECTIONS

CXCR3 and its ligands influence the TME by regulating angiogenesis, recruiting activated immune cells and affecting tumor cells in divergent roles either by promoting or inhibiting tumor progression. In addition to inducing chemotactic migration, CXCR3 ligands cause expansion of CD4 and CD8 T cells to induce Th1 polarization and persuade inflammation. The function of CXCR3 ligands is to attract T cells, co-stimulate their proliferation, differentiation, and activation suggesting that these ligands are important for priming T cell responses. CXCR3 induced response have therapeutic implications in peripheral inflammation and the TME. Thus CXCR3 ligands that attract effector lymphocytes into the tumor site can serve as therapeutic agents for colorectal as well as for many cancer models.

## References

[1] Moser B, Wolf M, Walz A, Loetscher P (2004). Chemokines: multiple levels of leukocyte migration control. Trends Immunol.

[2] Zlotnik A, Yoshie O (2012). The chemokine superfamily revisited. Immunity.

[3] Nomiyama H, Osada N, Yoshie O (2010). The evolution of mammalian chemokine genes. Cytokine Growth Factor Rev.

[4] Griffith JW, Sokol CL, Luster AD (2014). Chemokines and chemokine receptors: positioning cells for host defense and immunity. Annu Rev Immunol.

[5] Charo IF, Ransohoff RM (2006). The many roles of chemokines and chemokine receptors in inflammation. N Engl J Med.

[6] Murakami T, Kawada K, Iwamoto M, Akagami M, Hida K, Nakanishi Y, Kanda K, Kawada M, Seno H, Taketo MM, Sakai Y (2013). The role of CXCR3 and CXCR4 in colorectal cancer metastasis. Int J Cancer.

[7] Groom JR, Luster AD (2011). CXCR3 in T cell function. Exp Cell Res.

[8] Luster AD, Ravetch JV (1987). Biochemical characterization of a gamma interferon-inducible cytokine (IP-10). J Exp Med.

[9] Farber JM (1997). Mig and IP-10: CXC chemokines that target lymphocytes. J Leukoc Biol.

[10] Gasperini S, Marchi M, Calzetti F, Laudanna C, Vicentini L, Olsen H, Murphy M, Liao F, Farber J, Cassatella MA (1999). Gene expression and production of the monokine induced by IFN-gamma (MIG), IFN-inducible T cell alpha chemoattractant (I-TAC), and IFN-gamma-inducible protein-10 (IP-10) chemokines by human neutrophils. J Immunol.

[11] Sauty A, Dziejman M, Taha RA, Iarossi AS, Neote K, Garcia-Zepeda EA, Hamid Q, Luster AD (1999). The T cell-specific CXC chemokines IP-10, Mig, and I-TAC are expressed by activated human bronchial epithelial cells. J Immunol.

[12] Kolios G, Wright KL, Jordan NJ, Leithead JB, Robertson DA, Westwick J (1999). C-X-C and C-C chemokine expression and secretion by the human colonic epithelial cell line, HT-29: differential effect of T lymphocyte-derived cytokines. Eur J Immunol.

[13] Agace WW, Roberts AI, Wu L, Greineder C, Ebert EC, Parker CM (2000). Human intestinal lamina propria and intraepithelial lymphocytes express receptors specific for chemokines induced by inflammation. Eur J Immunol.

[14] Hamilton NH, Banyer JL, Hapel AJ, Mahalingam S, Ramsay AJ, Ramshaw IA, Thomson SA (2002). IFN-gamma regulates murine interferon-inducible T cell alpha chemokine (I-TAC) expression in dendritic cell lines and during experimental autoimmune encephalomyelitis (EAE). Scand J Immunol.

[15] Bonecchi R, Bianchi G, Bordignon PP, D’Ambrosio D, Lang R, Borsatti A, Sozzani S, Allavena P, Gray PA, Mantovani A, Sinigaglia F (1998). Differential expression of chemokine receptors and chemotactic responsiveness of type 1 T helper cells (Th1s) and Th2s. J Exp Med.

[16] Loetscher P, Pellegrino A, Gong JH, Mattioli I, Loetscher M, Bardi G, Baggiolini M, Clark-Lewis I (2001). The ligands of CXC chemokine receptor 3, I-TAC, Mig, and IP10, are natural antagonists for CCR3. J Biol Chem.

[17] Lu B, Humbles A, Bota D, Gerard C, Moser B, Soler D, Luster AD, Gerard NP (1999). Structure and function of the murine chemokine receptor CXCR3. Eur J Immunol.

[18] Cole KE, Strick CA, Paradis TJ, Ogborne KT, Loetscher M, Gladue RP, Lin W, Boyd JG, Moser B, Wood DE, Sahagan BG, Neote K (1998). Interferon-inducible T cell alpha chemoattractant (I-TAC): a novel non-ELR CXC chemokine with potent activity on activated T cells through selective high affinity binding to CXCR3. J Exp Med.

[19] Luster AD, Greenberg SM, Leder P (1995). The IP-10 chemokine binds to a specific cell surface heparan sulfate site shared with platelet factor 4 and inhibits endothelial cell proliferation. J Exp Med.

[20] Soejima K, Rollins BJ (2001). A functional IFN-gamma-inducible protein-10/CXCL10-specific receptor expressed by epithelial and endothelial cells that is neither CXCR3 nor glycosaminoglycan. J Immunol.

[21] Singh UP, Venkataraman C, Singh R, Lillard JW (2007). CXCR3 axis: role in inflammatory bowel disease and its therapeutic implication. Endocr Metab Immune Disord Drug Targets.

[22] Nishioji K, Okanoue T, Itoh Y, Narumi S, Sakamoto M, Nakamura H, Morita A, Kashima K (2001). Increase of chemokine interferon-inducible protein-10 (IP-10) in the serum of patients with autoimmune liver diseases and increase of its mRNA expression in hepatocytes. Clin Exp Immunol.

[23] Kabashima H, Yoneda M, Nagata K, Hirofuji T, Maeda K (2002). The presence of chemokine (MCP-1, MIP-1alpha, MIP-1beta, IP-10, RANTES)-positive cells and chemokine receptor (CCR5, CXCR3)-positive cells in inflamed human gingival tissues. Cytokine.

[24] Xia MQ, Bacskai BJ, Knowles RB, Qin SX, Hyman BT (2000). Expression of the chemokine receptor CXCR3 on neurons and the elevated expression of its ligand IP-10 in reactive astrocytes: *in vitro* ERK1/2 activation and role in Alzheimer’s disease. J Neuroimmunol.

[25] Teruya-Feldstein J, Tosato G, Jaffe ES (2000). The role of chemokines in Hodgkin’s disease. Leuk Lymphoma.

[26] Ohshima K, Tutiya T, Yamaguchi T, Suzuki K, Suzumiya J, Kawasaki C, Haraoka S, Kikuchi M (2002). Infiltration of Th1 and Th2 lymphocytes around Hodgkin and Reed-Sternberg (H&RS) cells in Hodgkin disease: relation with expression of CXC and CC chemokines on H&RS cells. Int J Cancer.

[27] Liu MT, Armstrong D, Hamilton TA, Lane TE (2001). Expression of Mig (monokine induced by interferon-gamma) is important in T lymphocyte recruitment and host defense following viral infection of the central nervous system. J Immunol.

[28] Romagnani P, Rotondi M, Lazzeri E, Lasagni L, Francalanci M, Buonamano A, Milani S, Vitti P, Chiovato L, Tonacchera M, Bellastella A, Serio M (2002). Expression of IP-10/CXCL10 and MIG/CXCL9 in the thyroid and increased levels of IP-10/CXCL10 in the serum of patients with recent-onset Graves’ disease. Am J Pathol.

[29] Medoff BD, Sauty A, Tager AM, Maclean JA, Smith RN, Mathew A, Dufour JH, Luster AD (2002). IFN-gamma-inducible protein 10 (CXCL10) contributes to airway hyperreactivity and airway inflammation in a mouse model of asthma. J Immunol.

[30] Schuh JM, Blease K, Hogaboam CM (2002). CXCR2 is necessary for the development and persistence of chronic fungal asthma in mice. J Immunol.

[31] Romagnani P, Beltrame C, Annunziato F, Lasagni L, Luconi M, Galli G, Cosmi L, Maggi E, Salvadori M, Pupilli C, Serio M (1999). Role for interactions between IP-10/Mig and CXCR3 in proliferative glomerulonephritis. J Am Soc Nephrol.

[32] Salmaggi A, Gelati M, Dufour A, Corsini E, Pagano S, Baccalini R, Ferrero E, Scabini S, Silei V, Ciusani E, De Rossi M (2002). Expression and modulation of IFN-gamma-inducible chemokines (IP-10, Mig, and I-TAC) in human brain endothelium and astrocytes: possible relevance for the immune invasion of the central nervous system and the pathogenesis of multiple sclerosis. J Interferon Cytokine Res.

[33] Belperio JA, Keane MP, Burdick MD, Lynch JP, Xue YY, Li K, Ross DJ, Strieter RM (2002). Critical role for CXCR3 chemokine biology in the pathogenesis of bronchiolitis obliterans syndrome. J Immunol.

[34] Agostini C, Cassatella M, Zambello R, Trentin L, Gasperini S, Perin A, Piazza F, Siviero M, Facco M, Dziejman M, Chilosi M, Qin S, Luster AD, Semenzato G (1998). Involvement of the IP-10 chemokine in sarcoid granulomatous reactions. J Immunol.

[35] Flier J, Boorsma DM, van Beek PJ, Nieboer C, Stoof TJ, Willemze R, Tensen CP (2001). Differential expression of CXCR3 targeting chemokines CXCL10, CXCL9, and CXCL11 in different types of skin inflammation. J Pathol.

[36] Davidson NJ, Leach MW, Fort MM, Thompson-Snipes L, Kühn R, Müller W, Berg DJ, Rennick DM (1996). T helper cell 1-type CD4+ T cells, but not B cells, mediate colitis in interleukin 10-deficient mice. J Exp Med.

[37] Pizarro TT, Arseneau KO, Cominelli F (2000). Lessons from genetically engineered animal models XI. Novel mouse models to study pathogenic mechanisms of Crohn’s disease. Am J Physiol Gastrointest Liver Physiol.

[38] Yuan YH, ten Hove T, The FO, Slors JF, van Deventer SJ, te Velde AA (2001). Chemokine receptor CXCR3 expression in inflammatory bowel disease. Inflamm Bowel Dis.

[39] Singh UP, Singh S, Taub DD, Lillard JW (2003). Inhibition of IFN-gamma-inducible protein-10 abrogates colitis in IL-10-/- mice. J Immunol.

[40] Uguccioni M, Gionchetti P, Robbiani DF, Rizzello F, Peruzzo S, Campieri M, Baggiolini M (1999). Increased expression of IP-10, IL-8, MCP-1, and MCP-3 in ulcerative colitis. Am J Pathol.

[41] Walz A, Schmutz P, Mueller C, Schnyder-Candrian S (1997). Regulation and function of the CXC chemokine ENA-78 in monocytes and its role in disease. J Leukoc Biol.

[42] Soto H, Wang W, Strieter RM, Copeland NG, Gilbert DJ, Jenkins NA, Hedrick J, Zlotnik A (1998). The CC chemokine 6Ckine binds the CXC chemokine receptor CXCR3. Proc Natl Acad Sci USA.

[43] Shibahara T, Wilcox JN, Couse T, Madara JL (2001). Characterization of epithelial chemoattractants for human intestinal intraepithelial lymphocytes. Gastroenterology.

[44] Scheerens H, Hessel E, de Waal-Malefyt R, Leach MW, Rennick D (2001). Characterization of chemokines and chemokine receptors in two murine models of inflammatory bowel disease: IL-10-/- mice and Rag-2-/- mice reconstituted with CD4+CD45RBhigh T cells. Eur J Immunol.

[45] Singh UP, Singh S, Iqbal N, Weaver CT, McGhee JR, Lillard JW (2003). IFN-gamma-inducible chemokines enhance adaptive immunity and colitis. J Interferon Cytokine Res.

[46] Jemal A, Siegel R, Ward E, Hao Y, Xu J, Murray T, Thun MJ (2008). Cancer statistics, 2008. CA Cancer J Clin.

[47] Rustgi AK (2007). The genetics of hereditary colon cancer. Genes Dev.

[48] Ekbom A, Helmick C, Zack M, Adami HO (1990). Ulcerative colitis and colorectal cancer. A population-based study. N Engl J Med.

[49] Munkholm P (2003). Review article: the incidence and prevalence of colorectal cancer in inflammatory bowel disease. Aliment Pharmacol Ther.

[50] Kawada K, Hosogi H, Sonoshita M, Sakashita H, Manabe T, Shimahara Y, Sakai Y, Takabayashi A, Oshima M, Taketo MM (2007). Chemokine receptor CXCR3 promotes colon cancer metastasis to lymph nodes. Oncogene.

[51] Garziera M, Toffoli G (2014). Inhibition of host immune response in colorectal cancer: human leukocyte antigen-G and beyond. World J Gastroenterol.

[52] Mihm MC, Mulé JJ (2015). Reflections on the Histopathology of Tumor-Infiltrating Lymphocytes in Melanoma and the Host Immune Response. Cancer Immunol Res.

[53] Flynn S, Stockinger B (2003). Tumor and CD4 T-cell interactions: tumor escape as result of reciprocal inactivation. Blood.

[54] den Boer AT, van Mierlo GJ, Fransen MF, Melief CJ, Offringa R, Toes RE (2005). CD4+ T cells are able to promote tumor growth through inhibition of tumor-specific CD8+ T-cell responses in tumor-bearing hosts. Cancer Res.

[55] Wang D, Dubois RN, Richmond A (2009). The role of chemokines in intestinal inflammation and cancer. Curr Opin Pharmacol.

[56] Duraiswamy J, Kaluza KM, Freeman GJ, Coukos G (2013). Dual blockade of PD-1 and CTLA-4 combined with tumor vaccine effectively restores T-cell rejection function in tumors. Cancer Res.

[57] Madorsky Rowdo FP, Baron A, Urrutia M, Mordoh J (2015). Immunotherapy in Cancer: A Combat between Tumors and the Immune System; You Win Some, You Lose Some. Front Immunol.

[58] Singh UP, Singh NP, Singh B, Hofseth LJ, Price RL, Nagarkatti M, Nagarkatti PS (2010). Resveratrol (trans-3,5,4′-trihydroxystilbene) induces silent mating type information regulation-1 and down-regulates nuclear transcription factor-kappaB activation to abrogate dextran sulfate sodium-induced colitis. J Pharmacol Exp Ther.

[59] Wang RH, Zheng Y, Kim HS, Xu X, Cao L, Luhasen T, Lee MH, Xiao C, Vassilopoulos A, Chen W, Gardner K, Man YG, Hung MC (2008). Interplay among BRCA1, SIRT1, and Survivin during BRCA1-associated tumorigenesis. Mol Cell.

[60] Chaudhary B, Elkord E, Regulatory T (2016). Regulatory T Cells in the Tumor Microenvironment and Cancer Progression: Role and Therapeutic Targeting. Vaccines (Basel).

[61] Wijtmans M, Verzijl D, Leurs R, de Esch IJ, Smit MJ (2008). Towards small-molecule CXCR3 ligands with clinical potential. ChemMedChem.

[62] Oelkrug C, Ramage JM (2014). Enhancement of T cell recruitment and infiltration into tumours. Clin Exp Immunol.

[63] Li K, Zhu Z, Luo J, Fang J, Zhou H, Hu M, Maskey N, Yang G (2015). Impact of chemokine receptor CXCR3 on tumor-infiltrating lymphocyte recruitment associated with favorable prognosis in advanced gastric cancer. Int J Clin Exp Pathol.

[64] Gajewski TF, Schreiber H, Fu YX (2013). Innate and adaptive immune cells in the tumor microenvironment. Nat Immunol.

[65] McDermott DF, Atkins MB (2013). PD-1 as a potential target in cancer therapy. Cancer Med.

[66] Peng W, Liu C, Xu C, Lou Y, Chen J, Yang Y, Yagita H, Overwijk WW, Lizée G, Radvanyi L, Hwu P (2012). PD-1 blockade enhances T-cell migration to tumors by elevating IFN-γ inducible chemokines. Cancer Res.

[67] Chow MT, Luster AD (2014). Chemokines in cancer. Cancer Immunol Res.

